# Response to AAEM’s “Response to the Yale PA Residency Program”

**DOI:** 10.5811/westjem.2021.6.53820

**Published:** 2022-01-01

**Authors:** Alina Tsyrulnik, Katja Goldflam, Ryan Coughlin, Jessica Bod, Sharon Chekijian, David Della-Giustina

**Affiliations:** Yale School of Medicine, Department of Emergency Medicine, New Haven, Connecticut

Dear Editor:

We appreciate the opportunity to respond to the letter to the editor in reference to our prior publications^1^,^2^ and to clarify the concerns raised. It seems that we, the authors of the original article, and the author(s) of the most recent letter to the editor have common ground on many of the issues presented. We believe that all emergency patients should be cared for by emergency physician-led teams. We agree that the training of our physician residents cannot be compromised.

First and foremost, we are proud of all our trainees and of the collaborative educational programs we have developed. As we noted previously, our original publication^1^ was “a brief innovation report,” meant to describe an educational innovation, and not intended to be interpreted as a comment on workforce challenges facing our specialty. In our original manuscript, we did not try to equate the graduating physician resident with a graduating physician assistant (PA) from our program but to make a comparison of milestone achievements after a year of postgraduate training. This point is made more explicit in the response to our original article: “We do not seek to equate the two programs or the skills of their respective graduates.”

The author(s) of the most recent letter to the editor claim that we promote PAs as “independent providers of patient care” in our manuscript. In fact, this language is from the background section of the paper and is quoting references^3^,^4^ to establish the importance of postgraduate training for PAs. Our clarifying letter to the editor explicitly states the following: “1. Advanced practice providers (APP) in emergency medicine should work with the supervision of an emergency medicine (EM) specialty-trained physician. 2. Patients should be cared for by emergency physician-led teams in the emergency department.”^2^

The author(s) of the letter to the editor state that “More than 25 states now allow nurse practitioners (NP) to care for patients without any physician involvement. This trend has now started for physician assistants (PA)...” While this deserves discussion and attention by relevant professional organizations and legislators, we do not see it as a mandate to halt educational programs. As educators, we believe that continuing medical education of all team members is essential to the safe care of patients regardless of professional degree.

The author(s) of the letter to the editor claim that the American Academy of Emergency Medicine (AAEM) and the American College of Emergency Physicians (ACEP) advocate an end to all postgraduate training programs for non-physicians. They cite an open letter published by a single author.^5^ In fact, the ACEP “Workforce of the Future”^6^ presentation lists “Support Standardized Training and Certification for APPs Working in the ED” as one of the ways to move forward in addressing the workforce issue facing EM. The Society of Academic Emergency Medicine (SAEM) and the American Board of Emergency Medicine (ABEM) are the lead organizations for this proposal.

An important detail we would like to address is the name of our program. Our original manuscript was submitted on July 13, 2020. Several organizations including ACEP and AAEM later called for the use of the term “resident” to be used strictly in reference to MD trainees. We have complied with this recommendation and our program has been renamed the “Yale Emergency Medicine APP Post-Graduate Training Program.”

As physician educators, we agree that the training of our EM residents should not be compromised. As is pointed out in our response publication, “due to our high ED patient volume, including multiple training sites, our physician trainees have not had a decrease in patient or procedure exposure.”^2^ Caseloads and procedure logs are monitored carefully to ensure ample exposure and opportunity for our EM residents on a continuous basis.

The author(s) of the letter to the editor accurately note that many critically ill patients are seen by PAs and NPs in the ED. While we agree with the AAEM position that emergency patients should be cared for by board-certified emergency physician-led teams,^7^ this does not negate the fact that all team members benefit from further training. The letter concludes this way: “the safety of our patients is at stake.” In that spirit, we hope we can all agree that a patient-centered approach to healthcare delivery is aided by comprehensive instead of minimal training of all members of a physician-led team.
